# A fast, sensitive and easy colorimetric assay for chitinase and cellulase activity detection

**DOI:** 10.1186/1754-6834-7-37

**Published:** 2014-03-10

**Authors:** Alessandro R Ferrari, Yasser Gaber, Marco W Fraaije

**Affiliations:** 1 Molecular Enzymology Group, Groningen Biomolecular Sciences and Biotechnology Institute, University of Groningen, Nijenborgh 4, 9747 AG, Groningen, The Netherlands; 2 Microbiology Department, Faculty of Pharmacy, Beni-Suef University, 62511 Beni-Suef, Egypt

**Keywords:** Chitinase, Cellulase, Chito-oligosaccharide oxidase, High-throughput screening, Chitin, DNS, Schales’ procedure, Cellulose, Colorimetric assay

## Abstract

**Background:**

Most of the current colorimetric methods for detection of chitinase or cellulase activities on the insoluble natural polymers chitin and cellulose depend on a chemical redox reaction. The reaction involves the reducing ends of the hydrolytic products. The Schales’ procedure and the 3,5-dinitrosalicylic acid (DNS) method are two examples that are commonly used. However, these methods lack sensitivity and present practical difficulties of usage in high-throughput screening assays as they require boiling or heating steps for color development.

**Results:**

We report a novel method for colorimetric detection of chitinase and cellulase activity. The assay is based on the use of two oxidases: wild-type chito-oligosaccharide oxidase, ChitO, and a mutant thereof, ChitO-Q268R. ChitO was used for chitinase, while ChitO-Q268R was used for cellulase activity detection. These oxidases release hydrogen peroxide upon the oxidation of chitinase- or cellulase-produced hydrolytic products. The hydrogen peroxide produced can be monitored using a second enzyme, horseradish peroxidase (HRP), and a chromogenic peroxidase substrate. The developed ChitO-based assay can detect chitinase activity as low as 10 μU within 15 minutes of assay time. Similarly, cellulase activity can be detected in the range of 6 to 375 mU. A linear response was observed when applying the ChitO-based assay for detecting individual chito-oligosaccharides and cello-oligosaccharides. The detection limits for these compounds ranged from 5 to 25 μM. In contrast to the other commonly used methods, the Schales’ procedure and the DNS method, no boiling or heating is needed in the ChitO-based assays. The method was also evaluated for detecting hydrolytic activity on biomass-derived substrates, that is, wheat straw as a source of cellulose and shrimp shells as a source of chitin.

**Conclusion:**

The ChitO-based assay has clear advantages for the detection of chitinase and cellulase activity over the conventional Schales’ procedure and DNS method. The detection limit is lower and there is no requirement for harsh conditions for the development of the signal. The assay also involves fewer and easier handling steps. There is no need for boiling to develop the color and results are available within 15 minutes. These aforementioned features render this newly developed assay method highly suitable for applications in biorefinery-related research.

## Background

Enzymatic degradation of cellulose and chitin is a hot research topic due to its potential for efficient utilization of the energy and carbon content of these polymers [1]. Chitin and cellulose are highly abundant and natural polymers of 1,4-β-linked sugar units of either N-acetyl-D-glucosamine or D-glucose, respectively. Chitin and cellulose share similarities in both structure and the enzymatic degradation mechanism. Generally, four groups of enzymes interact in the polymer degradation process: 1) exoenzymes, which are active on both ends of the polymer chain; 2) endoenzymes, which attack easily accessible glycosidic bonds or amorphous regions in the polymer chain; 3) dimer hydrolases, that is, β-glucosidases or chitobiosidase, which hydrolyze oligosaccharides; and 4) lytic polysaccharide monooxygenases, which introduce breaks in the crystalline region of the polymer chain and facilitate polymer unpacking [2-4]. A final mixture of monomeric, dimeric and oligomeric carbohydrate units is produced, which is commonly utilized for detection purposes. Using the reducing end functionalities in this mixture, a reaction with redox reagents develops a measurable color.

For detection of chitinolytic or cellulolytic activities, both soluble and insoluble substrates either natural or chemically modified are used. For example, assessment of chitinase activity can be accomplished with solubilized substrates such as ethylene glycol chitin, carboxymethyl chitin and 6-O-hydroxypropyl-chitin, or insoluble modified chitin substrates such as chitin azure and tritium-labeled chitin [2,5]. However, the use of native unmodified substrates is highly preferred compared to the use of surrogate substrates that are chemically modified. To monitor the enzymatic activity, the reducing sugars released by the action of enzymes are determined colorimetrically. The common colorimetric methods currently used for measuring the reducing sugar content are the 3,5-dinitrosalicylic acid (DNS) method and the ferricyanide-based Schales’ procedure [4,6,7]. The reduction of inorganic oxidants such as ferricyanide or cupric ions by the aldehyde/hemiacetal groups of the reducing sugar ends leads to color change that can be measured spectrophotometrically. However, there are several drawbacks of these methods such as: 1) use of alkaline medium which destroys part of the reducing sugars; 2) the necessity for heating or boiling for color development; 3) the long reaction time; 4) insensitivity at lower range of sugar concentrations; and 5) difficulty in use in high-throughput screening [8,9].

Chito-oligosaccharide oxidase (ChitO) identified in the genome of *Fusarium graminearum* was the first discovered oxidase capable of the oxidation of chito-oligosaccharides [10,11]. The oxidation takes place at the substrate C1 hydroxyl moiety leading to formation of equimolar amounts of H_2_O_2_ and the corresponding lactone. The produced lactone hydrolyzes spontaneously to the corresponding aldonic acids. ChitO displays excellent activity on the substrates N-acetyl-D-glucosamine, chitobiose, chitotriose and chitotetraose with *k*_cat_ values of around 6 s^−1^ and K_M_ values below 10 mM (6.3, 0.30, 0.26 and 0.25 mM, respectively) [11]. The wild-type ChitO displays very poor activity towards cellulose-derived oligosaccharides. However, by a structure-inspired enzyme engineering approach, we have designed a mutant, ChitO-Q268R, which displays a much higher catalytic efficiency towards cello-oligosaccharides [11]. The mutant enzyme displays *k*_cat_ values of around 7 s^−1^ for glucose, cellobiose, cellotriose and cellotetraose, while the K_M_ values vary to some extent (182, 22, 6.5 and 20 mM, respectively) [11]. The ChitO-Q268R displays a poor catalytic efficiency for the chito-oligosaccharides. With these two oxidase variants, ChitO (selective for N-acetyl-glucosamine derivatives) and ChitO-Q268R (selective for glucose derivatives), it is feasible to efficiently oxidize chitin- or cellulose-derived hydrolytic products. This inspired us to explore the use of ChitO for assay development.

In the current report we present a ChitO-based assay by which chitinase and cellulase activities can be detected in a quick, sensitive and facile method. The approach takes advantage of the hydrogen peroxide generated by ChitO or ChitO-Q268R when acting on products formed by hydrolytic activity of chitinases or cellulases, respectively. The well-established horseradish peroxidase (HRP) colorimetric assay was used for the detection of the produced H_2_O_2_. The use of these oxidases in combination with HRP constitutes a fast and sensitive method to detect chitinase and cellulase activity, without the necessity of a boiling step, commonly employed in other methods.

## Results and discussion

### ChitO-based assay and Schales’ method for chitinase detection

**Figure 1:**
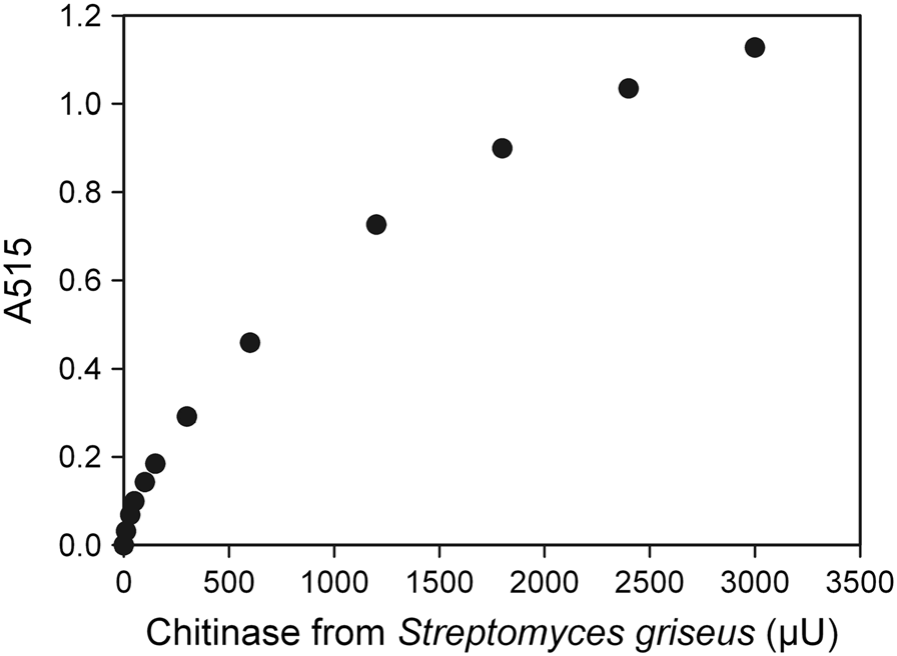
**Application of the ChitO-based assay for detection of the hydrolytic products of chitinase using colloid chitin as a substrate.** The average of the absorbance values at 515 nm of the triplicates was subtracted from the averaged blank value and plotted. ChitO, chito-oligosaccharide oxidase.

The chitinase ChitO-based assay is based on the oxidation of the chito-oligosaccharides by ChitO, which are formed by the action of the chitinases on the chitin. Upon oxidation of these substrates, a stoichiometric amount of H_2_O_2_ is produced by reduction of molecular oxygen. The hydrogen peroxide is used by HRP to convert 4-aminoantipyrine (AAP) and 3,5-dichloro-2-hydroxybenzenesulfonic acid (DCHBS) into a pink and stable compound [12]. As a result, the intensity of the pink color is proportional to the concentration of the available ChitO substrates. To test our assay for the detection of chitinase activity, a chitinase from *Streptomyces griseus* and colloid chitin as a substrate were used. Colloidal chitin is a natural unmodified substrate, easy to prepare and convenient for pipetting compared to chitin flakes. Varying amounts of chitinase were incubated with colloid chitin for 60 minutes to allow degradation of the chitin. Subsequently, the ChitO assay components (ChitO, AAP, DCHBS, and HRP) were added to the incubations in the 96-well microtiter plate. Development of a clear pink color is indicative of chitinase activity. By measuring the absorbance at 515 nm, the activity of ChitO, and hence the activity of chitinase, could be determined. A clear relationship was observed between the measured absorbance and increasing units of chitinase (Figure 1). In fact, the data shows a saturation curve which can be fitted with a simple hyperbolic formula:

$$A=A_{\max}.\left[\mathrm{chitinase}\right]/\left(x+\left[\mathrm{chitinase}\right]\right),R^{2}=0.996$$

**Figure 2:**
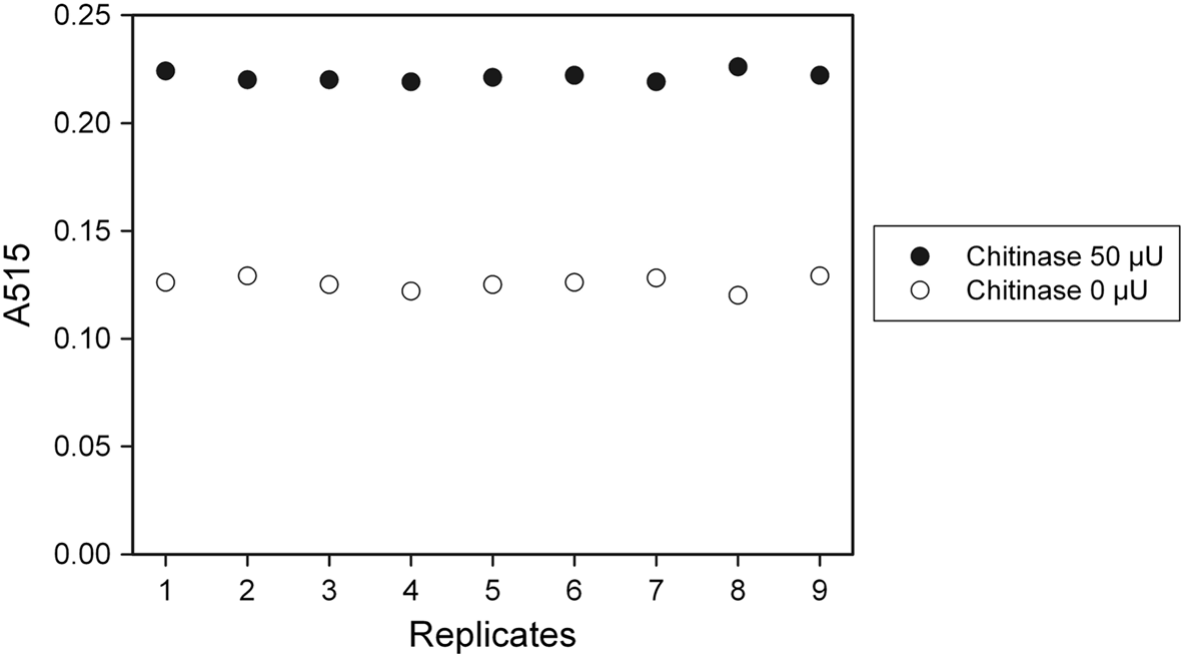
**Reproducibility of the ChitO-based assay with chitinase.** Absorbance values of the ChitO-based assay from nine replicates of colloidal chitin treated for 1 hour with 50 μU of chitinase from *Streptomyces griseus* were plotted against nine replicates of untreated colloidal chitin under the same assay conditions. ChitO, chito-oligosaccharide oxidase.

Interestingly, the assay could detect as low as 10 μU of chitinase with an assay time of only 15 minutes and using 0.12 U ChitO (*P* <1%). The blank reaction (colloidal chitin incubated without chitinase) revealed that colloidal chitin itself is a very poor substrate for ChitO. For such incubation a very weak signal (A_515_ = 0.12) was recorded and used as a blank. The reproducibility of the ChitO-based assay was assessed by comparing the corrected absorbance values on nine replicates of colloidal chitin treated with 50 μU of chitinase, to nine replicates of untreated colloidal chitin (Figure 2). The assay showed high reproducibility with a low standard deviation (<0.3%) for both samples.

**Figure 3:**
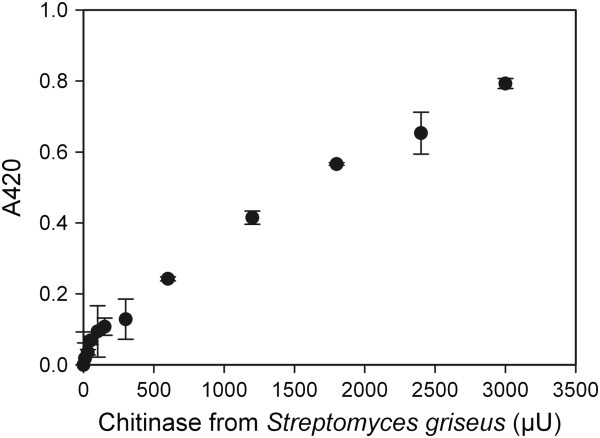
**Application of the Schales’ procedure to detect the hydrolytic products of chitinase using colloid chitin as a substrate.** For each sample, the average of the absorbance at 420 nm was subtracted from the averaged blank value and plotted.

**Figure 4:**
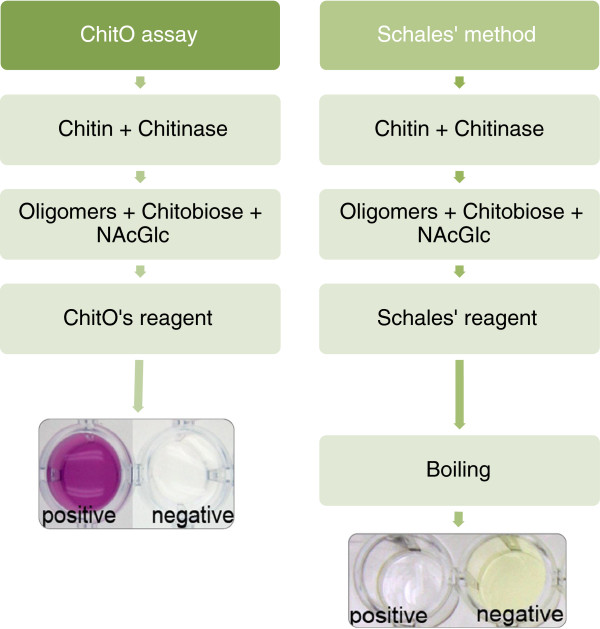
**Comparison of the outline of the Schales’ procedure and the developed ChitO-based assay.** Schales’ reagent, starting with a yellow color, reacts with the reducing sugars obtained from chitinase activity and after boiling a fading of the yellow color can be monitored at 420 nm. In the ChitO-based assay, the development of the pink product does not require any boiling step and is visible in a short time, depending on the concentration of oligomers in the reaction and the amount of ChitO used. Images edited to improve contrast. ChitO, chito-oligosaccharide oxidase. NAcGlc, N-acetyl-D-glucosamine.

For benchmarking, we compared the ChitO-based assay to the Schales’ procedure since it is one of the most common methods for the detection of chitinase activity [7,13]. The Schales’ reagent is yellow in color and reaction with reducing sugars results in color fading, which can be measured at 420 nm. Figure 3 shows the absorbance signal obtained in relation to the concentration of chitinase. A chitinase activity of 600 μU was found to be the lowest detection limit (*P* <3%). This value is 60 times higher than the detection limit of the ChitO-based assay (10 μU) indicating a higher sensitivity in favor of the ChitO assay. In addition, the recorded signal intensity of the ChitO assay was higher, by approximately two-fold, than Schales’ procedure. This can be concluded from comparing the signal responses in Figures 1 and 3, particularly when considering the range of 600 μU to 3,000 μU chitinase. It is important to note that the boiling step, an essential step in the Schales’ procedure, is omitted from the ChitO assay, which represents one of the main advantages (Figure 4).

### ChitO-based assay for cellulase detection

To adapt the ChitO-based assay for monitoring activity of cellulolytic enzymes, a ChitO mutant (ChitO-Q268R) was used instead of wild-type ChitO. ChitO-Q268R has a higher enzymatic efficiency towards glucose, cellobiose, cellotriose and cellotetraose compared to wild-type ChitO. We applied the assay using the same conditions as for detection of chitinase activity. As a model cellulase, an endocellulase from *Aspergillus niger* was used with a filter paper as a substrate. Endoglucanases typically hydrolyze accessible parts of the cellulose polymer and generate new chain ends. The generated cellotetraose and lower fragments will be substrates for ChitO-Q268R and consequently will allow H_2_O_2_ generation and development of the pink-colored product. The signal intensity, which is based on endocellulase activity, depends on the fraction of accessible β-glycosidic bonds in the substrate.

**Figure 5:**
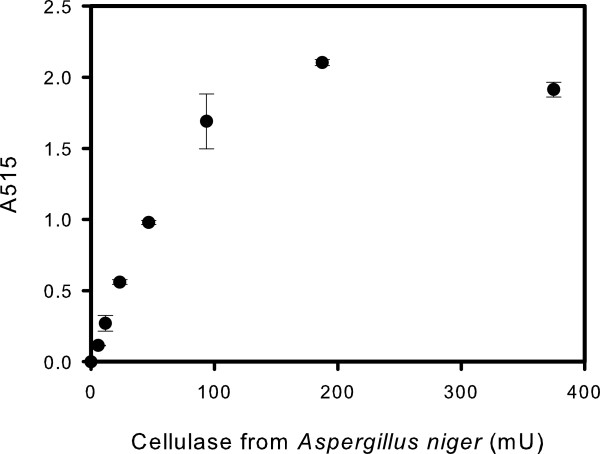
**Application of ChitO-based assay for the detection of the hydrolytic products of a cellulase from *Aspergillus niger *using filter paper as a substrate.** The mutant ChitO-Q268R was used instead of the wild-type ChitO. The average absorbance values at 515 nm of the triplicates were subtracted from the averaged blank value and plotted. ChitO, chito-oligosaccharide oxidase.

It was gratifying to see that using ChitO-Q268R in combination with HRP resulted in a clear and immediate color development. As was found for the ChitO-based chitinase assay, a directly proportional relationship of the absorbance to the amount of cellulase units was observed (Figure 5). The response curve started to level off when using >100 mU of the hydrolase. The lowest tested amount of endocellulase was 6 mU which could be detected with an assay time of 15 minutes using 0.13 U of ChitO-Q268R (*P* <0.5%). The commonly used colorimetric reagent to measure the cellulose saccharification is DNS [14]. There are many drawbacks of this method, such as non-reproducibility, complexity of reagents preparations and time-consuming. It also requires a strict control of temperature for proper color development and stability [15]. Moreover, the use of toxic reagents and phenolic compounds in large amounts makes it not a very environmentally-friendly method. Trials have been made to improve the DNS assay, such as reducing the amount of reagents used and adapting it to a microtiter plate assay. However, heating or boiling is still required in all of these approaches [16]. Both the DNS assay and the ChitO-based cellulase assay cannot distinguish between the contributions given by the different sugars present in the reaction mixture. However, the ChitO-based assay does not require alkaline medium and harsh treatment as in DNS, which results in degradation of the sugar content and decreased sensitivity [15,16]. On the whole, the assay represents a faster, high-throughput and ‘green’ method for cellulase detection when compared with the established DNS assay.

### ChitO-based assays: detecting defined substrates

**Figure 6:**
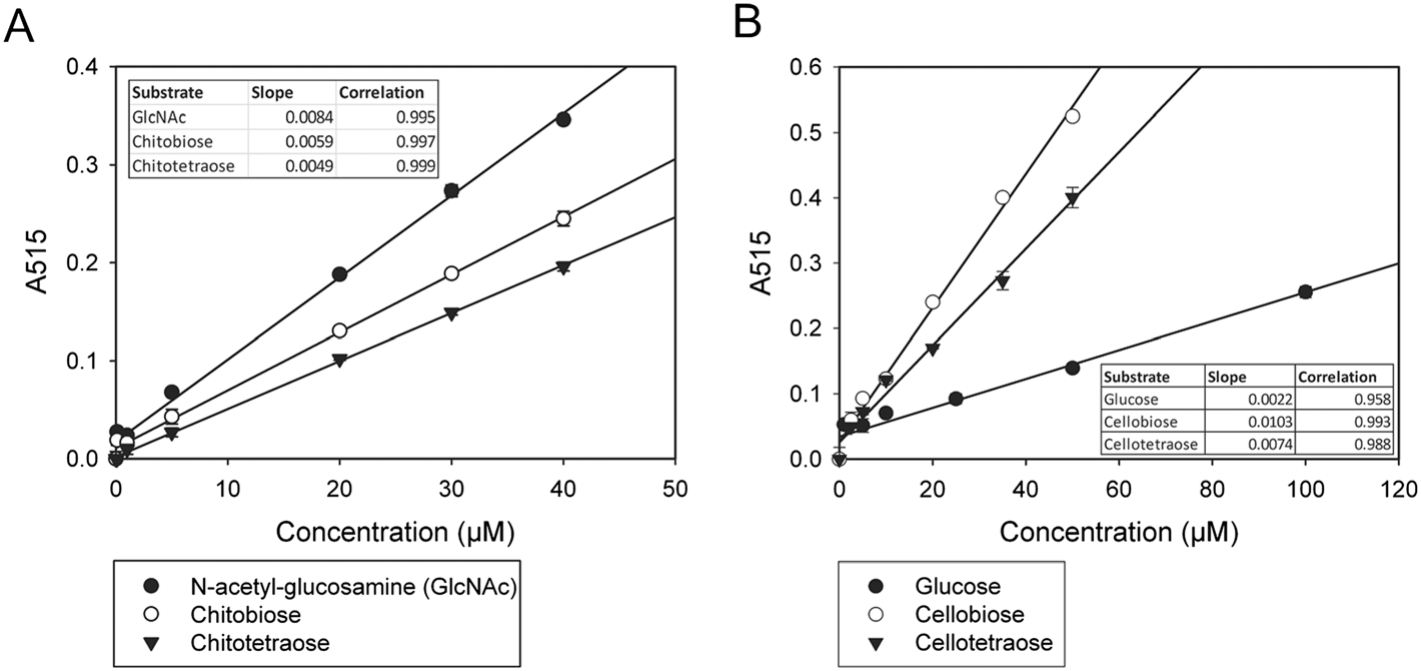
**Signal response of the ChitO-based assays.** Signal response of the ChitO-based assays when tested with varying concentrations of **(A)** N-acetyl-glucosamine, chitobiose and chitotetraose and **(B)** glucose, cellobiose and cellotetraose. ChitO, chito-oligosaccharide oxidase.

The color that develops in the aforementioned assay experiments is a sum of the ChitO activity on a mixture of different hydrolytic products produced by chitinase or cellulase activity. In order to identify the sensitivity of the assay for individual hydrolysis products, response curves were determined. Two sets of compounds were tested: chito-oligosaccharides and cello-oligosaccharides. The experiments were performed at pH 6 and 5, respectively, similar to the ChitO-chitinase and ChitO-cellulase detection experiments. Figure 6A shows a direct response of the signal when testing varying concentrations of N-acetyl-D-glucosamine, chitobiose and chitotetraose, representatives of the chitin degradation products. The limit of detection for N-acetyl-D-glucosamine, chitobiose and chitotetraose was 5 μM (*P* <5%). Based on the observed slopes, N-acetyl-D-glucosamine showed the highest signal response followed by chitobiose and chitotetraose. A similar trend has also been found with the Schales’ procedure and described in literature by Horn and Eijsink [9]. The second set of compounds tested represented cellulose degradation products: glucose, cellobiose and cellotetraose. Figure 6B shows a direct response of the ChitO assay signal to the increasing concentration of the compounds. The limit of detection was 25 μM for glucose and 10 μM for cellobiose and cellotetraose (*P* <5%). No specific trend of signal response to the compound’s length was observed. Cellobiose showed the highest signal response followed by cellotetraose and glucose.

### ChitO-based assays: monitoring hydrolysis of complex natural substrates

**Figure 7:**
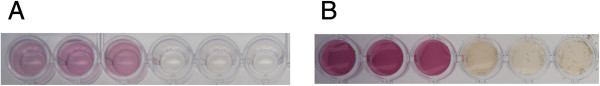
**Test of ChitO-based assay on real substrates. (A)** Shrimp shell treated with chitinase from *Streptomyces griseus* and **(B)** straw treated with cellulase from *Aspergillus niger*. Triplicates of the substrate treated with the hydrolase (left) are compared with triplicates of non-treated substrate (right). Images edited to improve contrast. ChitO, chito-oligosaccharide oxidase.

The ChitO assay showed applicability for detection of chitinase and cellulase activity on processed substrates such as colloid chitin and filter paper. We have tested the applicability of the assay on unprocessed and complex materials, that is, ground shrimp shell and wheat straw. In both cases a strong signal was observed (Figure 7). The assay was found to be very specific as the blank reactions did not yield any significant signal. The measured absorbance values for the triplicate samples showed only marginal differences, which confirms the above results of the assay reproducibility.

In the context of comparing the ChitO-based assay to the Schales’ procedure, the availability of reagents should also be addressed. The oxidases used in the ChitO-based assay are expressed in a standard expression system using *Escherichia coli* as host. The enzymes are stable at room temperature and active under the assay pH condition. A His-tag has been fused to the recombinant enzymes to facilitate the purification process. Expression in *E. coli* and subsequent purification can yield 40 mg (170 U) of purified protein per liter of culture [11]. Considering the low amount of ChitO used in the present experiments (0.12 U per sample), a 1 L culture provides sufficient ChitO for assaying over 1,400 samples.

Several strategies can be foreseen for further development of the ChitO-based assay. The formation of H_2_O_2_, that is a reactive oxidative species, can be used for detection by highly sensitive techniques. For example, an amperometric redox polymer-based biosensor replacing the colorimetric reagents can be utilized as has been done for cellobiose dehydrogenase [17]. Alternatively, the use of a fluorescent dye such as Amplex Red for H_2_O_2_ detections will enable the ChitO-based assay to work in real-time analyses and turbid materials such as soil samples. The present study has shown the applicability of ChitO-based assay for cellulose or chitinase activity detection. However, it can also find a potential application in the food industry, for example, for the detection of chitin and chitosan content in edible mushrooms [18].

## Conclusion

We have developed ChitO-based assays that are very sensitive in detecting chitinase or cellulase activities. The method allows the chromogenic detection of 10 μU of chitinase activity and 6 mU of endocellulase activity in just 15 minutes. The heating or boiling steps required for the Schales’ procedure or DNS method are not necessary, which renders the ChitO-based method extremely easy. It was also demonstrated that the method can be used to detect chitin- or cellulose-derived carbohydrates and monitoring the hydrolysis of complex natural materials. The assay is highly suitable for high-throughput approaches and its versatility makes it a powerful tool for the discovery, engineering or optimizing of enzymes involved in the field of biorefinery research.

## Methods

### Chemicals

Chitinase from *S. griseus*, HRP, cellulase from *A. niger*, N-acetyl-D-glucosamine and 3,5-dichloro-2-hydroxybenzenesulfonic acid sodium salt were purchased from Sigma-Aldrich (St Louis, MO, USA). One unit of HRP is defined as the amount of enzyme that will form 1.0 mg of purpurogallin from pyrogallol in 20 seconds at pH 6.0 at 20°C. One unit of chitinase is defined as the amount of enzyme that liberates 1.0 mg of N-acetyl-D-glucosamine from chitin per hour at pH 6.0 at 25°C in a 2-hour assay. One unit of cellulase is defined as the amount of enzyme that liberates 1.0 μmol of glucose from cellulose in 1 hour at pH 5.0 at 37°C. 4-Aminoantipyrine was purchased from Acros Organics (Geel, Belgium), D-glucose monohydrate was purchased from Merck (Darmstadt, Germany) and cellotetraose, chitobiose and chitotetraose were purchased from Dextra (Reading, UK). Cellobiose (purity >98%) was purchased from TCI Europe (Zwijndrecht, Belgium). Whatman filter paper grade 1 was purchased from GE Healthcare Life Sciences (Little Chalfont, UK). *E. coli* ORIGAMI2 DE3 was purchased from EMD Millipore (Billerica, MA, USA) and pET-SUMO vector was obtained from Invitrogen (Carlsbad, CA, USA).

### Colloidal chitin preparation

Colloidal chitin was prepared according to Shen *et al*. [8]. Briefly, 4.0 g of chitin was suspended in 37% HCl for 50 minutes and then 1.0 L of distilled water was slowly added. The colloid was centrifuged and the pellet washed with distilled water several times and then sterilized by autoclaving. Before each experiment, the amount of colloidal chitin to use was washed three times with MilliQ water and then resuspended in 50 mM phosphate buffer, reaching a pH value around 6.0.

### ChitO production and purification

The protein expression and purification was based on the methods previously described by Heuts *et al*. [11] with some modifications. Briefly, ChitO and ChitO-Q268R encoding genes were cloned in the pET-SUMO vector resulting in the expression of fusion proteins with a polyhistidine and a SUMO tag at the N-terminal end. Expression was carried out in *E. coli* ORIGAMI2 DE3 (EMD Millipore) for 69 hours at 17°C after which cells were harvested and sonicated in lysis buffer (50 mM Tris/HCl pH 7.6; 0.5 M NaCl; 10 mM imidazole). After ultra-centrifugation, the cell-free extract was incubated for 1 hour with 1.0 mL of Ni Sepharose (GE Healthcare) pre-equilibrated with lysis buffer. After washing with increasing concentrations of imidazole, the proteins were eluted with 0.5 M of imidazole. The samples were de-salted through Econo-Pac 10DG desalting columns (Bio-Rad, Hercules, CA, USA) and concentrated with Amicon Ultra (EMD Millipore). The protein concentration was determined as previously described [11]. One unit of wild-type ChitO is defined as the amount of enzyme that catalyzes the conversion of 1 μmol of chitobiose per minute. One unit of ChitO-Q268R is defined as the amount of enzyme that catalyzes the conversion of 1 μmol of cellobiose per minute.

### ChitO-based assay for chitinase detection

Increasing units of chitinase solution (10 μU to 3,000 μU) were incubated with colloid chitin, 3.0 mg/mL final concentration, in a 96-well microtiter plate to a final volume of 200 μL. The reactions were buffered with 50 mM KPi pH 6.0 and were kept at 30°C on a shaking incubator for 1 hour. The 96-well microtiter plate was briefly centrifuged at 4°C and 100 μL of supernatant was transferred to a new plate. Then, the ChitO assay components were added to the supernatant in the following order: 20 μL AAP (1 mM), 20 μL DCHBS (10 mM), 4 μL HRP (200 U/mL), 6 μL ChitO (20 U/mL) and 50 μL of 50 mM KPi at pH 6.0 to reach a volume of 200 μL. The assay was incubated for 15 minutes at room temperature to allow the formation of the pink product. All measurements were run in triplicates. The plates were read for absorbance at 515 nm. The values were corrected for both the path length and the blank (substrate in buffer) and the means of each triplicate were plotted. Samples not treated with chitinase were used as negative control. In order to rule out the probability of continuous enzymatic chitinase activity during the assay, the signal intensity from boiled samples were compared to non-boiled samples and the signal difference was found to be statistically insignificant.

### Schales’ procedure for chitinase detection

A series of increasing units of chitinase solutions were incubated with colloid chitin in a 96-well microtiter plate in a similar setting to the ChitO assay described above. The microtiter plate was briefly centrifuged at 4°C and 100 μL of supernatant was transferred in a new plate. A volume of 100 μL of Schales’ reagent (a solution of 0.5 M sodium carbonate and 0.5 g/L potassium ferricyanide in water) was added. The plate covered in aluminum foil was incubated at 100°C for 15 minutes and, after cooling down, read for absorbance at 420 nm. As a positive control, 50 mM of N-acetyl-D-glucosamine was used.

### ChitO-based assay for cellulase detection

Whatman filter paper number 1 was used as a substrate for the cellulase activity detection. The filter paper was cut into 0.5 cm diameter discs with a common office hole punch and accommodated on the bottom of the 96-well microtiter plate. The cellulase from *A. niger* was dissolved in 50 mM sodium citrate buffer pH 5.0 in different amounts (6 mU to 375 mU) and 200 μL of the solution was incubated with filter paper for 1 hour at 37°C in a shaking incubator. The microtiter plate was briefly centrifuged at 4°C and 100 μL of supernatant was transferred to a new plate. For detection of cellulose activity or detecting cellulose-derived sugars, the following components were added: 20 μL AAP (1 mM), 20 μL DCHBS (10 mM), 4 μL HRP (200 U/mL), 6 μL ChitO-Q268R (20 U/mL) and 50 μL of 50 mM KPi at pH 6.0 to reach a final volume of 200 μL. The assay was incubated for 15 minutes at room temperature to allow the formation of the pink product. All measurements were performed in triplicates. Samples not treated with cellulase were used as negative control. Similarly to ChitO-based chitinase assay experiments, a comparison of the signal obtained from boiled samples including cellulase to non-boiled samples showed no statistically significant difference.

### ChitO-based assay for detecting defined sugars

Two sets of compounds were used in the experiments. The first set comprised increasing concentrations (0.1 to 40 μM) of N-acetyl-D-glucosamine, chitobiose and chitotetraose. The second set comprised various concentrations of glucose (1 to 100 μM), cellobiose (2.5 to 50 μM) and cellotetraose (2.5 to 50 μM). A volume of 100 μL of compound solution was put in the well of the microtiter plate, followed by addition of ChitO or ChitO-Q268R reagents as described above to a final volume of 200 μL.

### ChitO-based assay for monitoring hydrolysis of complex natural substrates

Shrimps were purchased from the local market. The shells were peeled off, dried and blended in a common blender resulting in flocks of heterogeneous size. Wheat straw was purchased from a local supermarket, blended and sieved through a metallic sieve to obtain a small-particle powder. The ChitO assay was run in a 96-well microtiter plate using 10 mg of the ground shrimp shell or wheat straw as substrate and chitinase (29 mU) or cellulase (500 mU), respectively. The plates were incubated at 30°C and 37°C, respectively, for 1 hour. The plates were centrifuged and 100 μL of the supernatant was transferred to another plate. The color development using either ChitO or ChitO-Q268R reagents was performed as described above.

### Statistical analysis

Significance was determined according to the Student’s *t*-test using Excel software (Microsoft, Redmond, WA, USA). *p* values were accepted when <5%.

## Abbreviations

AAP: 4-Aminoantipyrine; ChitO: Chito-oligosaccharide oxidase; DCHBS: 3,5-Dichloro-2-hydroxybenzenesulfonic acid; DNS: 3,5-Dinitrosalicylic acid; HRP: Horseradish peroxidase.

## Competing interests

The authors declare that they have no competing interests.

## Authors’ contributions

MWF and ARF suggested the concept. ARF designed and performed the experiments and wrote the first draft of the manuscript. YG and MWF discussed the results and wrote the manuscript. All authors read and approved the final manuscript.
